# Oxidative stability of direct‐expanded chickpea–sorghum snacks

**DOI:** 10.1002/fsn3.1731

**Published:** 2020-07-16

**Authors:** Esayas K. Bekele, Matthew G. Nosworthy, Carol J. Henry, Phyllis J. Shand, Robert T. Tyler

**Affiliations:** ^1^ School of Nutrition, Food Science and Technology Hawassa University Hawassa Ethiopia; ^2^ College of Pharmacy and Nutrition University of Saskatchewan Saskatoon Saskatchewan Canada; ^3^ College of Agriculture and Bioresources University of Saskatchewan Saskatoon Saskatchewan Canada

**Keywords:** chickpea, extrusion, oxidation, sensory, shelf‐life, sorghum

## Abstract

In contrast to other pulses, chickpea has a relatively high fat content (3%–10%). This study was designed to investigate direct‐expanded chickpea–sorghum extruded snacks (50:50, 60:40, and 70:30 chickpea:sorghum, w/w) with respect to: their oxidative stability and sensory properties during accelerated (55°C) and room temperature (25°C) storage; correlations between chemical markers (peroxide value and *p*‐anisidine value) and sensory data during accelerated storage; and the shelf‐life of snacks extruded at the optimal expansion point as determined by a rotatable central composite design. Peroxide values and *p*‐anisidine values were in the range of 0–2.5 mEq/Kg and 5–30, respectively, for both accelerated and room temperature storage, and increased during storage (*p* < .05). 70:30 and 60:40 (w/w) chickpea–sorghum snacks had higher peroxide and *p*‐anisidine values compared to the 50:50 snack during storage at either temperature (*p* < .05). Rancid aroma and off‐flavor of 60:40 and 70:30 chickpea–sorghum snacks (slightly intense = 6) also were higher than that of the 50:50 snack (moderately weak = 3) (*p* < .05). Significant correlations (*p* < .05) were found between chemical markers and sensory attributes (*p* < .05). The study illustrated that shelf‐life decreased as the percentage of chickpea in the blend increased. Therefore, in terms of shelf‐life, a 50:50 chickpea–sorghum blend is preferable.

## Graphical abstract

The study investigated direct‐expanded chickpea–sorghum snacks (50:50, 60:40, and 70:30 chickpea:sorghum, w/w) with respect to oxidative stability during accelerated (55°C) and room temperature (25°C) storage as chickpea contains 3%–10% fat content on a dry weight basis. Both chemical and sensory analyses indicated that oxidative stability decreased as the percentage of chickpea in the blend increased. Therefore, in terms of shelf‐life, a 50:50 chickpea–sorghum blend is preferable.

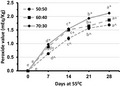

## 1 INTRODUCTION

Ready‐to‐eat (RTE) foods are intended for consumption without further heating or processing. High‐temperature extrusion is one technique for producing RTE foods and involves mixing, kneading, cooking, compressing, and forcing a molten material under high pressure through a small opening or die. Direct‐expanded extruded snacks are RTE products characterized by their puffed texture. A high expansion index and low apparent density are desirable properties of most direct‐expanded extruded snacks. A variety of plant‐based ingredients, including chickpea, have been used in the production of direct‐expanded extruded snacks (Obradovic, Babic, Subaric, Ackar, & Jozinovic, 2014).

Chickpea (*Cicer arietinum* L.) belongs to the Fabaceae family and is a rich source of complex carbohydrate, protein, vitamins, and minerals (Costa, Queiroz‐Monici, Reis, & Oliveira, 2006). The protein content of chickpea ranges from 19% to 27%, carbohydrate content from 52% to 71% (Hall, Hillen, & Robinson, 2017), and oil content from 3% to 10% (Gul, Egesel, & Turhan, 2008) on a dry weight basis. The oil content of chickpea is higher than that of most other pulses such as lentil (1.1%), red kidney bean (1.1%), field pea (1.3%), brown bean (1.4%), and turtle bean (1.6%) (Wang & Daun, 2004). The fatty acid profile of chickpea oil has been reported as palmitic acid (8%–12%), palmitoleic acid (1%), stearic acid (1%–5%), oleic acid (24%–43%), linoleic acid (42%–57%), and linolenic acid (2%–4%) (Dandachy, Mawlawi, & Obeid, 2019; Jukanti, Gaur, Gowda, & Chibbar, 2012).

Sorghum (*Sorghum bicolor* L.) belongs to the family Poaceae, and its protein content ranges from 9% to 17%, carbohydrate content from 77% to 89%, and lipid content from 2% to 6% on a dry weight basis (Palavecino, Penci, Calderon‐Dominguez, & Ribotta, 2016). The fatty acid profile of sorghum oil has been reported as palmitic acid (12%–15%), palmitoleic acid (1%), stearic acid (1%–3%), oleic acid (34%–37%), linoleic acid (42%–43%), and linolenic acid (1%–2%) (Afify, Hossam, Samiha, & Azza, 2012; Zhang et al., 2019). Sorghum is used for human food in Africa, Asia, and other semi‐arid regions of the world. In contrast, in the United States and Australia, sorghum is cultivated primarily for animal feed but usage as human food is increasing (Stefoska‐Needham, Beck, Johnson, & Tapsell, 2015).

The practice of developing nutritious extruded snacks by blending different ingredients has increased as the preference for nutritious snacks has increased. However, a better understanding of the stability of snacks developed from blended ingredients is important as it affects shelf‐life and nutrient content (Yadav, Singh, & Arora, 2018). Lipid oxidation is one of the principal causes of the loss of nutritional and organoleptic quality of foods during storage and a major determinant of shelf‐life. Extruded products are highly susceptible to oxidation due to their low water activity and high interfacial surface area as the material is highly porous (Barden & Decker, 2016). Even a low level of fat may cause problems related to oxidation. However, the oxidative stability during storage of lipid in extruded snacks containing chickpea has not been examined. Hence, the objectives of this study were to investigate oxidative stability and undertake descriptive sensory analysis of direct‐expanded chickpea–sorghum snacks during accelerated (55°C) and room temperature (25°C) storage; examine correlations between chemical markers and sensory data during accelerated storage; and determine the shelf‐life of chickpea–sorghum snacks. Storage at room temperature was investigated as it is a practical storage and distribution temperature for extruded snacks. Accelerated storage trials at 55°C also were conducted to determine their usefulness for estimating the shelf‐life of direct‐expanded chickpea–sorghum snacks (Ng, Anderson, Coker, & Ondrus, 2007).

## 2 MATERIALS AND METHODS

### 2.1 Raw materials

Kabuli chickpea (500 kg) and sorghum (500 kg) were purchased from Diefenbaker Spice & Pulse (Elbow, SK, Canada) and Sinner Bros. & Bresnahan Food Inc. (Casselton, ND, USA), respectively. Glacial acetic acid, chloroform, methanol, sodium hydroxide, hydrochloric acid (37% w/w), and starch indicator (1% w/v aqueous solution) were purchased from Fisher Scientific (Ottawa, ON, Canada). Isooctane (2, 2, 4‐trimethylpentane), potassium dichromate, potassium iodide, *p*‐anisidine, sodium thiosulfate, chymotrypsin (from bovine pancreas 4129 Type II, lyophilized powder, P40 units/mg protein), trypsin (from bovine pancreas 4129 Type IX‐S, lyophilized powder, 13,000–20,000 BAEE units/mg), and protease (from *Streptomyces grisseus* Type XIV, P3.5 units/mg) were purchased from Sigma‐Aldrich (Oakville, ON, Canada). All reagents were of analytical grade.

### 2.2 Sample preparation

#### 2.2.1 Blend ratio determination

Chickpea and sorghum grain were milled at the Saskatchewan Food Industry Development Centre Inc. (Saskatoon, SK, Canada) using a hammer mill (Model DAO6, The Fitzpatrick Company, Elmhurst, IL, USA) having a screen size of 0.8 mm. The particle size of the ground material ranged from 0.1 to 0.8 mm. To determine the optimal blend ratio, the in vitro protein digestibility (IVPD) and amino acid composition of raw chickpea and sorghum flours were determined as described previously by House, Hill, Neufeld, Franczyk, and Nosworthy (2019).

The IVPD and amino acid composition of 10:90, 20:80, 30:70, 40:60, 50:50, 60:40, 70:30, 80:20, and 90:10, w/w, chickpea–sorghum blends were determined mathematically based on the experimentally determined IVPD and amino acid composition of raw chickpea and sorghum flours. The amino acid scores of the raw flours and blends were determined by comparing their amino acid compositions to that of the reference pattern specified by the United States Food and Drug Administration (CFR 21CFR101.9), which is that of a 2‐ to 5‐year‐old child (FAO/WHO, 1991). The IVPD Corrected Amino Acid Scores (IVPDCAAS) of the raw flours and the blends were determined by multiplying IVPD and the lowest amino acid score, as described by House et al. (2019). The blend ratios for chickpea–sorghum snacks were selected on the basis of theoretical IVPD corrected amino acid scores (IVPDCAAS). The 70:30 chickpea–sorghum blend was identified as where the IVPDCAAS reached a plateau and therefore was chosen for this study. For the purpose of determining the effect of blending on lipid oxidation and shelf‐life, chickpea–sorghum snacks having blend ratios of 50:50 and 60:40 also were considered in the study. IVPDCAAS values for raw sorghum and raw chickpea were 27% and 74%, respectively. Calculated IVPDCAAS values for raw 50:50, 60:40, and 70:30 chickpea–sorghum blends were 58, 65, and 73%, respectively.

#### 2.2.2 Extrusion

The chickpea–sorghum blends were mixed using a Dmx Quad Action 500^TM^ blender (Daniels Food Equipment, Parkers Prairie, MN, USA). Extrusion was performed using a corotating, twin‐screw extruder (model EV‐32; Clextral, Firminy, France) equipped with a volumetric feeder (Clextral VF/40/25‐2) and a two‐blade die face cutter, at the Saskatchewan Food Industry Development Centre Inc. The barrel length, screw diameter, and die diameter of the extruder were 768, 32, and 2.7 mm, respectively. The extruder barrel had six zones. The temperatures of zones 1, 2, and 3 were set at 40°C, 80°C, and 120°C, respectively; the last three zones were kept at the same temperature, which was varied between 111°C and 169°C based on a rotatable central composite design having two center points. Feed moisture content was varied between 15% and 21%. Screw speed and feed rate were maintained at 396 rpm and 26 kg/hr, respectively. Each sample was processed in duplicate under each processing condition. Expansion index (EI) was determined according to the method of Meng, Threinen, Hansen, and Driedger (2010). EI measurements were taken 10 times on extrudates from each processing run and averaged. Based on surface model regression analysis of the EI, the maximal expansion point for all blends was found to be at 169°C barrel temperature and 15% feed moisture. Snacks from each of the three blend ratios produced at the maximal expansion point were used for the lipid oxidative stability study and were stored at −80°C until analyzed.

### 2.3 Sample storage for the lipid oxidative stability study

Accelerated and room temperature sample storage were conducted according to Ng et al. (2007). For each chickpea–sorghum snack (50:50, 60:40, and 70:30, w/w) produced at the maximal expansion point, four 100‐g samples were heat‐sealed in aluminum pouches (Sigma‐Aldrich) and stored at 55°C (accelerated) or 25°C (room temperature). Samples for accelerated storage were stored in an incubator (Forma Scientific, Marietta, OH, USA); samples were taken from the incubator for analysis at 7, 14, 21, and 28 days. Analysis of samples stored at room temperature was done every 28 days. The analysis was performed in quadruplicate.

### 2.4 Chemical analysis

The snacks were ground using a WonderMill™ grain mill (Pocatello, ID, USA) at bread setting.

The particle size of the ground material was less than 1 mm. Lipid was extracted using chloroform–methanol according to Folch, Lees, and Stanley (1957) with modifications. Flours were mixed with 2:1 (v/v) chloroform–methanol and agitated using a magnetic stirrer (Fisher Scientific) at a speed of 600 rpm for 20 min. Calcium chloride solution (0.001 M) was added to each sample with stirring. Each sample was filtered through No. 4 Whatman filter paper (Fisher Scientific), and the filtrates were centrifuged at 490*g* for 10 min. The upper phase was removed using a pipette and discarded. The lower chloroform layer was evaporated, and the residual lipid was used for chemical analysis. The peroxide and *p*‐anisidine values of the extracted oils were determined according to AOCS (1998) official methods Cd 8–53 and Cd 18–90, respectively. Protein and ash contents were determined according to AOAC (1997) official methods Ba 4e‐93 and Bc 5–49, respectively. Carbohydrate content was determined by difference (Honi, Mukisa, & Mongi, 2018).

### 2.5 Descriptive sensory analysis

#### 2.5.1 Selection and training of panelists

Eighteen panelists were recruited for sensory analysis through advertisements at the University of Saskatchewan, Saskatoon, SK, and via personal communication. The triangle test was carried out according to ISO 4120 (2004), and 13 panelists were selected based on their ability to select oxidized product. Six days of training was provided for the selected panelists. Training of panelists and final testing of snacks were carried out according to the generic descriptive sensory analysis method as described by Lawless and Heymann (2010). Chickpea–sorghum snacks: (a) stored at 65°C for 3 days; (b) frozen at −80°C and thawed at 25°C; and (c) stored at 65°C for 25 days, were used for the training sessions. During training, panelists identified and described perceivable product attributes, as well as attributes of reference standards on which the rating of the generated attributes was based. In cases where reference standards were not available, definitions were provided. Panelists also commented using a 10‐point scale ballot based on the selected descriptors. For monitoring, panelists were provided each day with six coded samples to evaluate. They were provided with 60:40 and 70:30 chickpea–sorghum snacks that had been (a) stored at 65°C for seven days and coded with three digits, (b) frozen at −80°C immediately after production and then thawed at 25°C and labeled as fresh, and (c) frozen at −80°C immediately after production and then thawed at 25°C and coded with three digits. The performance of panelists was determined on the basis of the scores provided while evaluating the samples. Panelists were ranked for each attribute based on the *F*‐value, and the top ten were selected for final descriptive sensory analysis.

#### 2.5.2 Sample testing

The final descriptive sensory analysis was performed by 10 trained panelists on extruded snacks stored under accelerated conditions. The sensory analysis was done at 0, 7, 14, 21, and 28 days of storage. Panelists were provided eight coded samples to evaluate (a) stored (55°C) samples of 50:50, 60:40, and 70:30 chickpea–sorghum extruded snacks, in duplicate, which were coded with three digits; (b) a fresh (stored at −80°C immediately after production) sample of the 70:30 chickpea–sorghum snack which was labeled as fresh; and (c) a fresh (stored at −80°C immediately after production) sample of the 70:30 chickpea–sorghum snack which was masked by coding with three digits. In addition to the samples, panelists were provided with freshwater and lemon water for rinsing between samples. Panelists were asked to evaluate the samples using the 10‐point scale (none = 0 to extremely intense = 10) on the ballot provided. The purpose of providing fresh samples was to monitor the reliability of the scores obtained from panelists.

### 2.6 Shelf‐life determination

To predict the shelf‐life of the chickpea–sorghum snacks, a zero‐order reaction for peroxide value was used (Andarwulan et al., 2014). The general formula for determining the order of reaction is as follows:

(1) $$dA/dt=k{\left(A\right)}^{n}$$

Integrating the above formula with *n* = 0 provides a zero‐order reaction formula:‐

(2) $$\left(dA/dt\right)=k{\left(A\right)}^{n}\left(n=0\right)$$

(3) $$A_{0}=A_{t}-kt$$

(4) $$t=(A_{t}-A_{0})/k$$

where *t*, *A_t_*, *A*_0,_ *k*, and *n* represent shelf‐life in days, peroxide value in mEq/kg at storage time t, peroxide value in mEq/kg at *t* = 0, the slope of the regression equation for peroxide value during storage in mEq/kg/day, and order of reaction, respectively.

### 2.7 Statistical analysis

Proximate composition was analyzed using one‐way ANOVA. Peroxide value, *p*‐anisidine value, and sensory data obtained for chickpea–sorghum snacks across the storage period were analyzed by two‐way ANOVA. Significant differences (*p* < .05) between means of the parameters were determined by Fisher LSD. Regression analysis was carried out to determine the maximal expansion points and relationships between chemical markers and sensory intensities, as well as to determine the shelf‐life of the snacks (Vik, 2014). Statgraphics Centurion version 18.1.12 (Statgraphics Technologies, Plains, VA, USA) was used for analysis.

## 3 RESULTS AND DISCUSSION

### 3.1 Proximate composition

The fat, protein, ash, and carbohydrate contents of raw chickpea were determined to be 7, 20, 2.5, and 71%, respectively, on a dry weight basis. Corresponding values determined for raw sorghum were 3, 10, 1.3, and 85% on a dry weight basis. Others have reported fat contents for chickpea and sorghum ranging from 3% to 10% and 2 to 6%, respectively, and protein contents ranging from 19% to 27% and 6 to 17%, respectively (Gul et al., 2008; Hall et al., 2017; Palavecino et al., 2016). The fat, protein, and carbohydrate contents differed (*p* < .05) among the extruded samples (Table 1). The fat and protein contents of extruded snacks increased (*p* < .05) as the ratio of chickpea in the blend increased, resulting in fat contents ranging from 5.1% to 5.9% and protein contents ranging from 15% to 17%.

**TABLE 1:** Proximate composition of direct‐expanded chickpea–sorghum snacks extruded at 169°C barrel temperature and 15% feed moisture, expressed on a dry weight basis

Sample	Protein (%)	Fat (%)	Ash (%)	Carbohydrate (%)
50:50 Chickpea–sorghum snack	15.29 ± 0.10^c^	5.06 ± 0.16^c^	1.94 ± 0.05^b^	77.72 ± 0.30^a^
60:40 Chickpea–sorghum snack	16.32 ± 0.04^b^	5.36 ± 0.08^b^	2.30 ± 0.14^a^	76.29 ± 0.01^b^
70:30 Chickpea–sorghum snack	17.46 ± 0.01^a^	5.91 ± 0.14^a^	2.15 ± 0.04^ab^	74.49 ± 0.19^c^

Note

Data are presented as mean ± *SD* on a dry weight basis (*n* = 4) and were analyzed via one‐way ANOVA and the Fisher LSD post hoc test. Samples with different letters in the same column are significantly different (*p* < .05).

### 3.2 Expansion index

Expansion index (EI) is the ratio of the extrudate diameter to the diameter of the extruder die. Regression analysis was carried out on the expansion indices of snacks obtained from extrusion of 50:50, 60:40, and 70:30 chickpea–sorghum blends at barrel temperatures ranging from 111 to 169°C and feed moistures ranging from 15% to 21%. The EIs of 50:50, 60:40, and 70:30 chickpea–sorghum snacks ranged from 3.00 to 3.98, 3.00 to 3.80, and 2.70 to 3.30, respectively. Expansion index was analyzed in order to determine the extrusion conditions which generated optimal expansion, as snacks prepared under these conditions would be used for the shelf‐life studies. Based on the regression results, temperature and moisture had significant (*p* < .05) effects on EI (Table 2). Temperature increased EI, whereas moisture had the opposite effect. An earlier study reported similar results (Lazou, Michailidis, Thymi, Krokida, & Bisharat, 2007). It was determined that for all chickpea–sorghum blends, snacks prepared at a barrel temperature of 169°C and a moisture content of 15% had higher EIs compared to those prepared at lower temperatures or higher moisture contents; hence, snacks prepared under these conditions were used for the subsequent shelf‐life studies.

**TABLE 2:** Regression coefficients for expansion index of direct‐expanded chickpea–sorghum snacks for several extrusion factors and their interactions

Terms	Coefficients		
	50:50 snack	60:40 snack	70:30 snack
A: Barrel temperature	0.09*	0.08*	0.13*
B: Feed Moisture	−0.28*	−0.23*	−0.13*
Constant	3.42*	3.43*	3.12*
AB	0.02	0.02	0.02
AA	0.03	0.04*	
BB	0.00	−0.04*	

Note

Significance of regression coefficients was determined based on ANOVA and Fisher's test (*n* = 4). The asterisk (*) indicates significant (*p* < .05) coefficients. The model R^2^ values 50:50, 60:40, and 70:30 chickpea–sorghum snacks were 98, 99, and 93%, respectively, significant at *p* < .05.

### 3.3 Peroxide and *p*‐anisidine values

**FIGURE 1:**
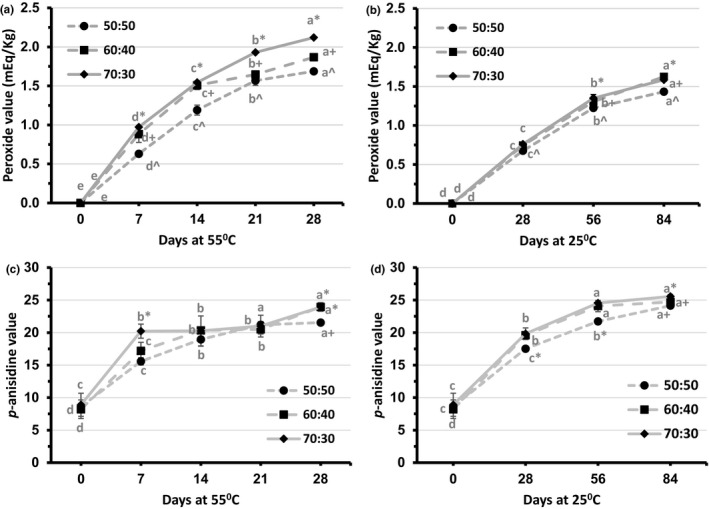
Peroxide values (a) and (b) and *p*‐anisidine values (c) and (d) of direct‐expanded chickpea–sorghum snacks stored at 55°C or 25°C. Data were analyzed via two‐way ANOVA with Fisher's test (*n* = 4). Significant (*p* < .05) differences between days, but within blend ratios, are designated by different letters. Significant (*p* < .05) differences between blend ratios, but within days, are designated by different nonalphanumeric characters

The oxidative stability of the lipid extracted from the extruded snacks was assessed using peroxide value (Figure 1a and b) and *p*‐anisidine value (Figure 1c and d). For both accelerated storage (55°C) and room temperature storage (25°C), peroxide values were not different (*p* > .05) between extruded blends at day 0. Over time, the peroxide values of chickpea–sorghum snacks increased (*p* < .05) under both storage conditions, indicating that lipid oxidation had occurred. Other studies have reported increases in peroxide value during storage of extrudates (Shahmohammadi, Bakar, Russly, Noranizan, & Mirhosseini, 2016; Shaviklo, Thorkelsson, Rafipour, & Sigurgisladottir, 2011). In the current study, the rate of peroxide development was faster in snacks stored at 55°C than at 25°C. Similarly, Lee, Lee, and Choe (2007) reported an enhancing effect of storage temperature on peroxide value during storage. Peroxide values for 70:30 and 60:40 chickpea–sorghum snacks were higher (*p* < .05) than for the 50:50 blend at all storage intervals, with the exception of day 0, under both storage conditions. This was attributed to the significantly higher fat contents of the 70:30 (5.9%) and 60:40 (5.4%) blends compared to the 50:50 (5.1%) blend. In addition, chickpea fat is higher in polyunsaturated fatty acids, linoleic acid in particular, compared to sorghum fat, making it more susceptible to oxidation (Jukanti et al., 2012; Stefoska‐Needham et al., 2015). Chickpea also has lower total phenolics and tannin contents as compared to sorghum, which further increases the vulnerability of chickpea fat to oxidation (Gaytan‐Martinez et al., 2017; Rani & Khabiruddin, 2016). The peroxide value of the 70:30 chickpea–sorghum snack was higher (*p* < .05) than that of the 60:40 blend at days 7, 14, 21, and 28 in the case of accelerated storage, and at day 56 in the case of room temperature storage. Again, this was to be expected due to the higher fat content of the 70:30 blend as mentioned above. However, the peroxide value of the 60:40 blend was higher (*p* < .05) than that of the 70:30 blend at day 84 under room temperature storage, but the difference was small and probably not of practical significance.

The peroxide values of 50:50, 60:40, and 70:30 chickpea–sorghum snacks stored under accelerated conditions ranged between 0 and 1.7, 0 and 1.9, and 0 and 2.1 mEq/kg, respectively, during the 28‐day storage period. In the case of room temperature storage, the corresponding peroxide values ranged between 0 and 1.4, 0 and 1.6, and 0 and 1.6 mEq/kg, respectively, over the 84‐day storage period. According to Codex (1999) and Canadian Food and Drug Regulations (2019), the safe peroxide limit is 10 mEq/kg, whereas according to the United States Food and Drug Administration (FDA, 2019), the safe limit is 5 mEq/kg. Therefore, peroxide values of snacks prepared from all three blends and stored at 55°C or 25°C were in the safe range throughout the study period, indicating that accelerated storage reflected storage at room temperature.

*p*‐anisidine values for 50:50, 60:40, and 70:30 chickpea–sorghum snacks ranged from 8.6 to 21.5, 8.2 to 23.9, and 8.9 to 23.9, respectively, for accelerated storage, and 8.6 to 24.1, 8.2 to 24.7, and 14.2 to 25.6, respectively, for room temperature storage (Figure 1c and d). *p*‐anisidine values were not different (*p* > .05) among extruded blends at day 0, but increased during storage (*p* < .05). These results are similar to those of a previous study where cookies prepared with margarine showed significant increments in *p*‐anisidine value during storage (Bialek, Rutkowska, Bialek, & Adamska, 2016). Fluctuation in *p*‐anisidine values can occur when the carboxylic acid group and C = C bond structures are involved in the formation of aldehydes, ketones, and alcohols that contribute to the *p*‐anisidine value (Sun‐Waterhouse, Thakorlal, & Zhou, 2011). This might explain why the *p*‐anisidine values for chickpea–sorghum snacks did not exhibit regular increments during accelerated storage. In the case of room temperature storage, however, the reaction rates of carboxylic acid groups and C = C bond structures would have been lower at the lower temperature.

In the case of accelerated storage, *p*‐anisidine values were different (*p* < .05) among 50:50, 60:40, and 70:30 chickpea–sorghum snacks at days 7 and 28. The *p*‐anisidine value of the 70:30 blend (20.2) was higher (*p* < .05) than that of the 50:50 (15.8) or the 60:40 (17.2) blend at day 7, whereas those of both the 60:40 (23.9) and 70:30 (23.9) blends were higher (*p* < .05) than that of the 50:50 blend (21.5) at day 28. As was the case for peroxide value, differences in the *p*‐anisidine value among 50:50, 60:40, and 70:30 chickpea–sorghum extruded snacks during storage would reflect differences in their fat contents and fatty acid profiles, as well as the total phenolics and tannin contents of the blends.

In the case of room temperature storage, the *p*‐anisidine values of 50:50, 60:40, and 70:30 chickpea–sorghum snacks increased (*p* < .05) during the storage period. The *p*‐anisidine values of 60:40 and 70:30 chickpea–sorghum blends showed increases with storage time until day 56 (*p* < .05). The *p*‐anisidine values of both the 60:40 and 70:30 blends were higher (*p* < .05) than that of the 50:50 blend at days 28 and 56. The rate of increase in the *p*‐anisidine value was higher in snacks stored under accelerated conditions than at room temperature. Lee et al. (2007) reported that the rate of increase in the *p*‐anisidine value increased with storage temperature, indicating both temperature and time dependence of lipid oxidation. The rate of increase in the *p*‐anisidine value began to level off during the latter part of the storage period in the current study. This might be due to declining levels of the most readily oxidizable fatty acids in the snacks, linolenic acid in particular.

### 3.4 Sensory analysis

**FIGURE 2:**
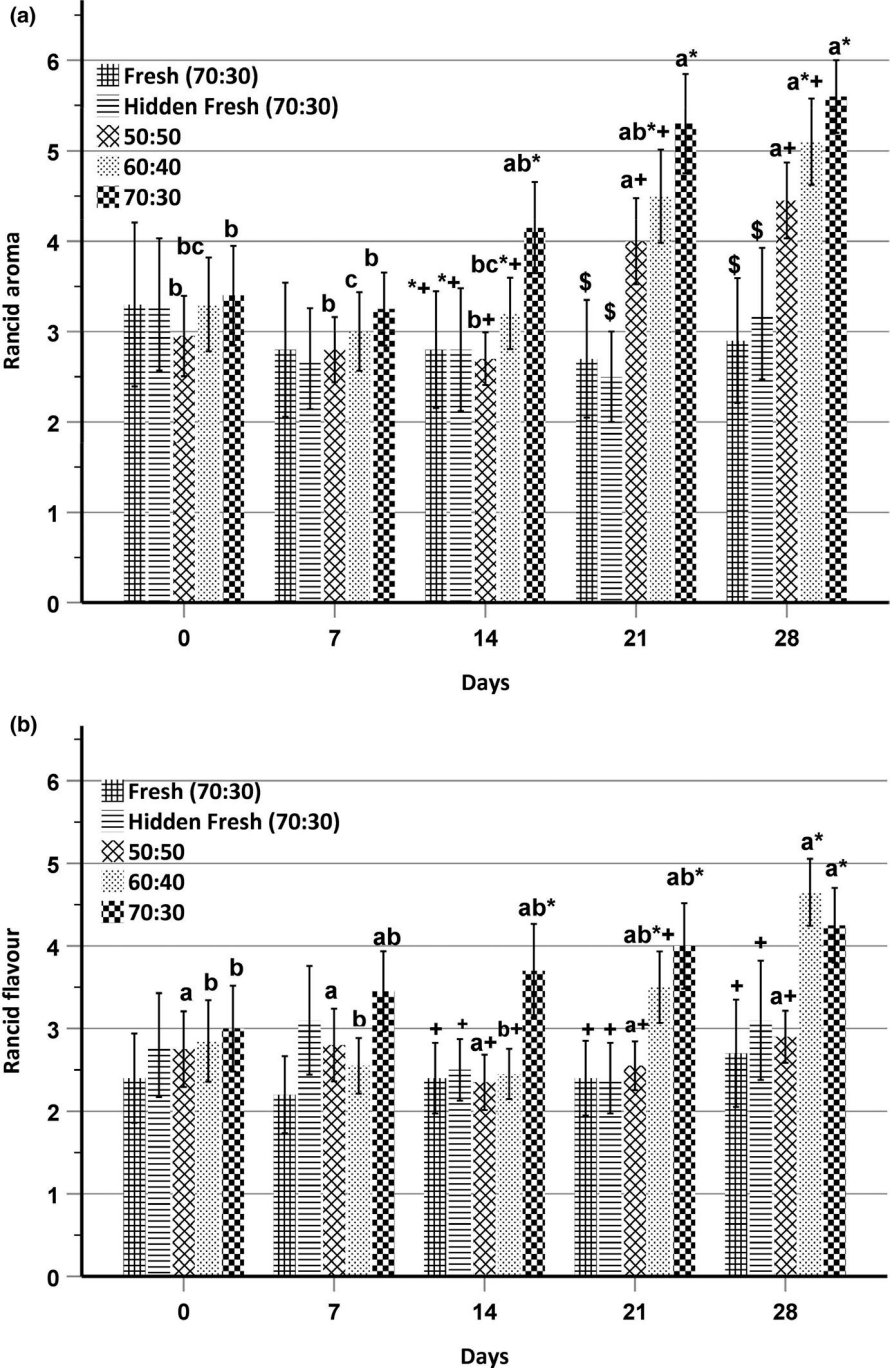
(a) Rancid aroma and (b) rancid flavor intensities for direct‐expanded chickpea–sorghum snacks stored at 55°C (*n* = 4). Data were analyzed using two‐way ANOVA with Fisher's test. Significant (*p* < .05) differences between days, but within blend ratios, are designated by different letters. Significant (*p* < .05) differences within days are designated by different nonalphanumeric characters. Intensity scale ranging from 0 = none to 10 = extremely intense was used. The number of panelists involved was 10

**FIGURE 3:**
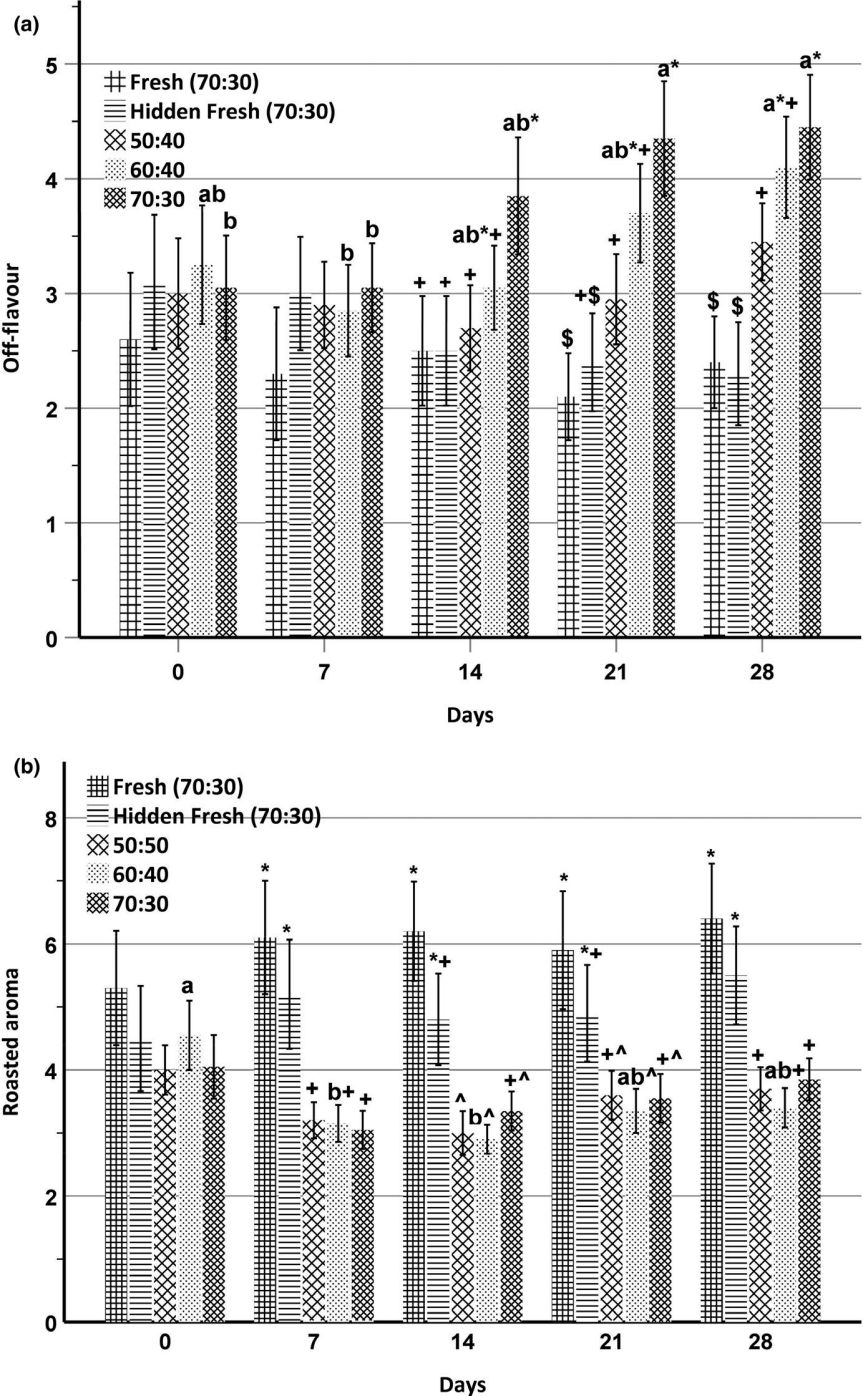
(a) Off‐flavor and (b) roasted aroma intensities for direct‐expanded chickpea–sorghum snacks stored at 55°C (*n* = 4). Data were analyzed using two‐way ANOVA with Fisher's test. Significant (*p* < .05) differences between days, but within blending ratios, are designated by different letters. Significant (*p* < .05) differences within days are designated by different nonalphanumeric characters. Intensity scale ranging from 0 = none to 10 = extremely intense was used. The number of panelists involved was 10

The reference standards and definitions used for sensory evaluation are listed in Table 3. Sensory evaluation was undertaken on snacks stored under accelerated conditions only, due to the time constraints of the trained panelists. Sensory attribute intensities over the storage period are described in Figures 2 and 3. Rancid aroma intensity scores for stored 50:50, 60:40, and 70:30 chickpea–sorghum snacks ranged from 3.0 to 4.5, 3.0 to 5.1, and 3.3 to 5.6, respectively (Figure 2a). Rancid aroma intensity was approximately 3.0 (moderately weak) for all samples at day 0 and day 7, but the rancid aroma intensities of stored 60:40 and 70:30 chickpea–sorghum snacks were significantly higher (*p* < .05) than that of the 50:50 chickpea–sorghum snack at days 14, 21, and 28. The rancid aroma intensity score of the 70:30 blend was 4.1 (slightly weak), 5.3 (neither intense nor weak), and 5.6 (slightly intense) at days 14, 21, and 28, respectively, whereas the corresponding scores for the 50:50 blend were 2.7 (moderately weak), 4.0 (slightly weak), and 4.5 (neither intense nor weak). The rancid aroma intensity scores for the 60:40 chickpea–sorghum blend at days 14, 21, and 28 were 3.2 (moderately weak), 4.5 (neither intense nor weak), and 5.1 (neither intense nor weak), respectively. During storage, the rancid aroma intensity scores for all chickpea–sorghum snacks were higher (*p* < .05) than those of the fresh samples, indicating advancement of lipid oxidation. Rancid aroma intensity was higher (*p* < .05) for all stored chickpea–sorghum snacks at days 21 and 28 as compared to days 0 and 7, again indicating the progression of lipid oxidation.

**TABLE 3:** Sensory attributes evaluated for direct‐expanded chickpea–sorghum snacks and corresponding reference standards and definitions employed

Attributes	Reference standards	Definitions^a^
Rancid aroma	Corn oil heated at 240°C for 10 min	Aroma of strongly oxidized oil
Rancid flavor	Corn oil heated at 240°C for 10 min	Flavor of strongly oxidized oil
Roasted aroma	Roasted chickpea	Aroma from roasted chickpea
Roasted flavor	Roasted chickpea/roasted barley	Aroma from roasted chickpea/barley
Off‐flavor	—	Presence of uncharacteristic flavor notes
Hardness	Cheesy puffs	Force applied by molar teeth to compress the food
Crispiness	Rice crisps	Louder and high‐pitched noise from food during mastication
Stickiness	Cheesy puffs	Degree of attachment to the teeth
Dissolvability	Cheesy puffs	Time the food stays in the mouth
Bitterness	Boiled coffee (1 g/5 ml)	Taste on the tongue associated with bitter solutions such as caffeine
Overall flavor	—	Any flavor notes coming from the food in the mouth

^a^ Adapted from Lawless and Heymann (2010).

Rancid flavor intensity scores for stored 50:50, 60:40, and 70:30 chickpea–sorghum snacks ranged from 2.3 to 2.9, 2.5 to 4.7, and 3.0 to 4.3, respectively (Figure 2b). While initially moderately weak for all samples, the rancid flavor intensity of the stored 70:30 snack (3.8) was higher (*p* < .05) than that of stored 50:50 (2.7) and 60:40 (3.1) chickpea–sorghum snacks and fresh samples (2.4) at day 14. At day 28, rancid flavor intensities of stored 60:40 (4.1) and 70:30 (4.5) snacks were higher (*p* < .05) than those of the stored 50:50 (2.9) chickpea–sorghum snack and fresh samples (2.7). Rancid flavor intensities of 60:40 and 70:30 chickpea–sorghum snacks on day 28 were higher (*p* < .05) than at day 0. Similar to this study, Rababah et al. (2011) reported significant development of rancidity and off‐flavor with storage time in extruded corn chips.

In the current study, off‐flavor intensity was not significant (*p* > .05) among extruded snacks at 0 days and 7 days of storage (Figure 3a). The stored 70:30 chickpea–sorghum snack scored higher (*p* < .05) in off‐flavor at days 14, 21, and 28 compared to the fresh sample, as well as the 50:50 chickpea–sorghum snack. On day 28, stored 50:50, 60:40, and 70:30 chickpea–sorghum snacks scored higher (*p* < .05) in off‐flavor compared to the fresh samples. The higher scores for rancid flavor and off‐flavor for 60:40 and 70:30 chickpea–sorghum snacks compared to the 50:50 snack during storage again reflected the fat contents of the snacks.

Initially, there were no differences in roasted aroma among the chickpea–sorghum snacks (Figure 3b). However, from day 7 to day 28, all stored chickpea–sorghum snacks scored lower (*p* < .05) in roasted aroma intensity than did fresh samples. Franklin et al. (2018) reported a reduction in roasted aroma intensity with storage time in roasted almonds, due perhaps to the increasing presence of volatiles arising from lipid oxidation.

There was no difference (*p* > .05) in stickiness, dissolvability, or bitterness (due to mono‐ and diglycerides) across samples or over time. Rancidity in stored snacks was detected by panelists even though the peroxide values of the snacks were below the regulated limit (5 mEq/Kg). Zajdenwerg, Branco, and Alamed (2011) reported that sensory detection of oxidized lipid in Brazil nut occurred before identification of changes in chemical markers. Overall, the 50:50 chickpea–sorghum snack was found to be the most stable based on sensory ratings provided by panelists.

### 3.5 Correlations

Linear and quadratic correlations between sensory attributes and chemical markers (peroxide value and *p*‐anisidine value) were determined. Both correlations were found to be significant (*p* < .05) in most cases. However, in a few cases, only quadratic correlations were significant. Therefore, quadratic correlations only are presented in Table 4. Peroxide values of 50:50, 60:40, and 70:30 chickpea–sorghum snacks showed significant (*p* < .05) positive correlations with the rancid aromas of the corresponding snacks, with *R* ^2^ values of .86, .69, and .93, respectively. Peroxide values of 60:40 and 70:30 snacks showed significant (*p* < .05) positive correlations with rancid flavor (*R* ^2^ = .72 and .73, respectively) and off‐flavor (*R* ^2^ = .74 and .80, respectively).

**TABLE 4:** Coefficients of determination (*R* ^2^) for quadratic correlations between sensory and chemical markers for direct‐expanded chickpea–sorghum snacks

	Off‐flavor			Rancid flavor			Roasted aroma			Rancid aroma			Peroxide		
	*a*	*b*	*c*	*a*	*b*	*c*	*a*	*b*	*c*	*a*	*b*	*c*	*a*	*b*	*c*
Dissolvability	0.65*	0.01	0.20	0.79	0.01	0.34	0.25	0.03	0.39	0.27	0.03	0.21	0.04	0.00	0.20
Bitterness	0.25	0.52	0.26	0.36	0.11	0.01	0.59*	0.24	0.06	0.42	0.25	0.00	0.63*	0.30	0.19
Overall flavor	0.71*	0.64*	0.60*	0.40	0.36	0.57	0.13	0.00	0.20	0.00	0.49	0.54	0.09	0.29	0.32
*p‐*anisidine	0.16	0.54	0.77*	0.05	0.78*	0.69*	0.45	0.87*	0.36	0.85*	0.53	0.83*	0.99*	0.87*	0.94*
Roasted flavor	0.13	0.08	0.31	0.33	0.07	0.41	0.13	0.62*	0.32	0.02	0.09	0.29	0.21	0.67	0.46
Peroxide	0.20	0.74*	0.80*	0.13	0.72*	0.73*	0.49	0.89*	0.71*	0.86*	0.69*	0.93*			
Rancid aroma	0.16	0.89*	0.88*	0.10	0.82*	0.71*	0.37	0.03	0.04						
Roasted aroma	0.26	0.45	0.36	0.71	0.26	0.18									
Rancid flavour	0.51	0.71*	0.59*												

Note

Chickpea–sorghum blends are represented as follows: *a* = 50:50, *b* = 60:40, *c* = 70:30. Significance of the coefficients was analyzed by ANOVA (*N* = 4) with Fisher's test. The asterisk (*) indicates significant correlation. The significance is *p* < .05.

The *p*‐anisidine values of 50:50 and 70:30 chickpea–sorghum snacks exhibited significant (*p* < .05) positive correlations with rancid aroma, with *R* ^2^ values of .85 and .83, respectively (Table 4). The *p*‐anisidine value also exhibited a significant (*p* < .05) positive correlation with rancid flavor of the 60:40 (*R* ^2^ = .78) and 70:30 (*R* ^2^ = .69) snacks, and off‐flavor of the 70:30 chickpea snack (*R* ^2^ = .77). Peroxide values and *p*‐anisidine values were found to be significantly (*p* < .05) and positively correlated for 50:50, 60:40, and 70:30 snacks, with *R* ^2^ values of .99, .87, and .94, respectively.

Zajdenwerg et al. (2011) reported that peroxide and *p*‐anisidine values had significant positive, linear correlations with oxidized aroma. This again indicates that trends in peroxide and *p*‐anisidine values can be indicative of what will happen to the intensity over time of sensory attributes such as rancidity and off‐flavor. Correlation analysis also was performed for the sensory attributes (Table 4). The rancid aroma of 60:40 and 70:30 chickpea–sorghum snacks was found to have a significant (*p* < .05) positive correlation with off‐flavor (*R* ^2^ = .89 and 0.88, respectively) and rancid flavor (*R* ^2^ = .82 and .71, respectively). Off‐flavor and rancid flavor of 60:40 and 70:30 snacks also exhibited significant (*p* < .05) positive correlations, with *R* ^2^ values of .71 and .59, respectively.

### 3.6 Determination of shelf‐life

Peroxide value was selected as an indicator of shelf‐life for the chickpea–sorghum snacks stored under accelerated conditions or at room temperature, since a maximum safe limit (5 mEq/Kg) is recognized by the United States Food and Drug Administration (FDA, 2019). Slopes of regression equations of peroxide values for 50:50, 60:40, and 70:30 chickpea–sorghum snacks stored at 55°C and 25°C were determined and used in rate Equation (4). The safe peroxide limit (5 mEq/kg) was substituted for peroxide value at storage time t, and an initial peroxide value of zero obtained from the peroxide analysis was substituted for peroxide value at storage t = 0. The slopes obtained from the regression equations for peroxide values and used in Equation (4) were 0.062, 0.064, and 0.074 for 50:50, 60:40, and 70:30 snacks, respectively, stored under accelerated conditions, and 0.017, 0.019, and 0.019, respectively, for the corresponding snacks stored at room temperature. The shelf‐lives of 50:50, 60:40, and 70:30 chickpea–sorghum snacks stored at room temperature (25°C) were determined to be 9.8, 8.8, and 8.8 months, respectively; corresponding shelf‐lives for accelerated storage (55°C) were determined to be 2.7, 2.6, and 2.3 months, respectively. The calculated shelf‐life of the 50:50 snack was higher (*p* < .05) than those of 60:40 and 70:30 snacks under accelerated conditions, whereas the shelf‐lives of both 50:50 and 60:40 snacks were higher (*p* < .05) than that of the 70:30 snack when stored at room temperature. This indicates that the 50:50 snack in general was more stable compared to the 60:40 and 70:30 snacks. According to Honi et al. (2018), extruded snacks made from orange‐fleshed sweet potato and Bambara groundnuts had shelf‐lives ranging from 4 to 5 months at room temperature depending on the percentage of groundnut used, which varied the total fat content from 6% to 12%. Similar to the current study, as the proportion of the higher fat component (Bambara nut) in the snack increased, the shelf‐life decreased. The longer shelf‐life of the chickpea–sorghum snacks stored at room temperature compared to extruded sweet potato/groundnut snacks (Honi et al., 2018) reflected differences in both fat content and degree of unsaturation. The shelf‐life of extruded products depends on both composition and storage conditions. In this study, the sensory score for rancid aroma intensity of chickpea–sorghum snacks stored under accelerated conditions was between 4 (moderately weak) and 6 (neither intense nor weak) on day 28 using a 10‐point scale. This indicates that the chickpea–sorghum snacks have a high probability of being rancid toward the end of the calculated shelf‐life (68–81 days); thus, the sensory data are in agreement with the experimentally determined shelf‐life values.

## 4 CONCLUSIONS

Through analysis of peroxide value, the shelf‐life during room temperature storage was calculated to be longer for the snack prepared from a 50:50 chickpea–sorghum blend than for snacks prepared from 60:40 and 70:30 blends. Shelf‐lives for snacks prepared from both 50:50 and 60:40 blends were longer than for snacks prepared from a 70:30 blend under accelerated storage conditions. Similarly, the *p*‐anisidine values for both storage conditions indicated that the 50:50 snack was more stable than that prepared from a 70:30 chickpea–sorghum blend. Trained sensory panelists provided corroborating evidence in that rancid aroma, rancid flavor, and off‐flavor increased during accelerated storage, but the increase was least for the 50:50 blend. These data indicate that whereas from a nutritional perspective, the optimal blend ratio for chickpea–sorghum snacks was 70:30 chickpea–sorghum, shelf‐life and consumer acceptability after storage were maximized at a blend ratio of 50:50. Accelerated storage testing was useful in reinforcing the results of room temperature storage in that the 50:50 chickpea–sorghum snack was more stable than that prepared from a 70:30 chickpea–sorghum blend. The study demonstrated the potential of using whole grain chickpea and whole grain sorghum blends for the production of direct‐expanded snacks having acceptable sensory and shelf‐life characteristics.

## CONFLICTS OF INTEREST

The authors declare no conflict of interest.

## AUTHOR CONTRIBUTIONS

Esayas Kinfe Bekele, Robert T. Tyler, and Carol J. Henry designed the study. Esayas Kinfe Bekele carried out the laboratory analysis and data analysis. Phyllis J. Shand provided advice and training with respect to sensory and statistical analysis. Esayas Kinfe Bekele, Matthew G. Nosworthy, Robert T. Tyler, and Carol J. Henry interpreted the data. All authors contributed to drafting the manuscript and approved the final version.

## ETHICAL APPROVAL

Ethical approval was obtained from the Biomedical Research Ethics Board of the University of Saskatchewan under ID number BIO16‐261.

## INFORMED CONSENT

Written informed consent was obtained from all study participants.
